# THE1B may have no role in human pregnancy due to ZNF430-mediated silencing

**DOI:** 10.1186/s13100-023-00294-6

**Published:** 2023-05-22

**Authors:** Zheng Zuo

**Affiliations:** Shenzhen University, Shenzhen, Guangdong China

**Keywords:** ZNF430, ZNF100, THE1B, THE1A, CRH

## Abstract

THE1-family retrovirus invaded the primate genome more than 40 million years ago. Dunn-Fletcher et al. reported one THE1B element upstream of CRH gene alters gestation length by upregulating corticotropin-releasing hormone expression in transgenic mice and concluded it has the same role in human as well. However, no promoter or enhancer mark has been detected around this CRH-proximal element in any human tissue or cell, so probably some anti-viral factor exists in primates to prevents it from wreaking havoc. Here I report two paralogous zinc finger genes, ZNF430 and ZNF100, that emerged during the simian lineage to specifically silence THE1B and THE1A, respectively. Contact residue changes in one finger confers each ZNF the unique ability to preferentially repress one THE1 sub-family over the other. The reported THE1B element contains an intact ZNF430 binding site, thus under the repression of ZNF430 in most tissues including placenta, it is questionable whether or not this retrovirus has any role in human pregnancy. Overall, this analysis highlights the need to study human retroviruses’ functions in suitable model system.

**Supplementary Information:**

The online version contains supplementary material available at 10.1186/s13100-023-00294-6.

## Background

In 2018, Dunn-Fletcher et al. [1] reported in PLOS Biology that when one simian-specific THE1B element upstream of human CRH gene was inserted into transgenic mice, it can upregulate CRH hormone expression and influence gestation length, so they concluded that this THE1B element functions as enhancer for CRH in human as well, though neither any canonical enhancer mark (H3K27ac, H3K4me3, DNase) nor significant THE1B-CRH fusion transcript has been detected in any human tissue/cell, as reported by the original paper and ENCODE database [2] (Fig. S1). In fact, to combat the invasion of endogenous retroviruses (ERVs), hundreds of KRAB-domain zinc fingers genes (KZNFs) emerged in the primate lineage and evolved to specifically recognize and silence different ERVs by depositing repressive H3K9me3 chromatin marks [3], thus their findings must be scrutinized more carefully in the context of co-evolution (or arm race) between ERVs and anti-viral KZNFs. If some primate-specific, anti-THE1B KZNF exists and functions in human placenta, the extrapolation of results in mice study to human is unwarranted.

## Main text

**Fig. 1:**
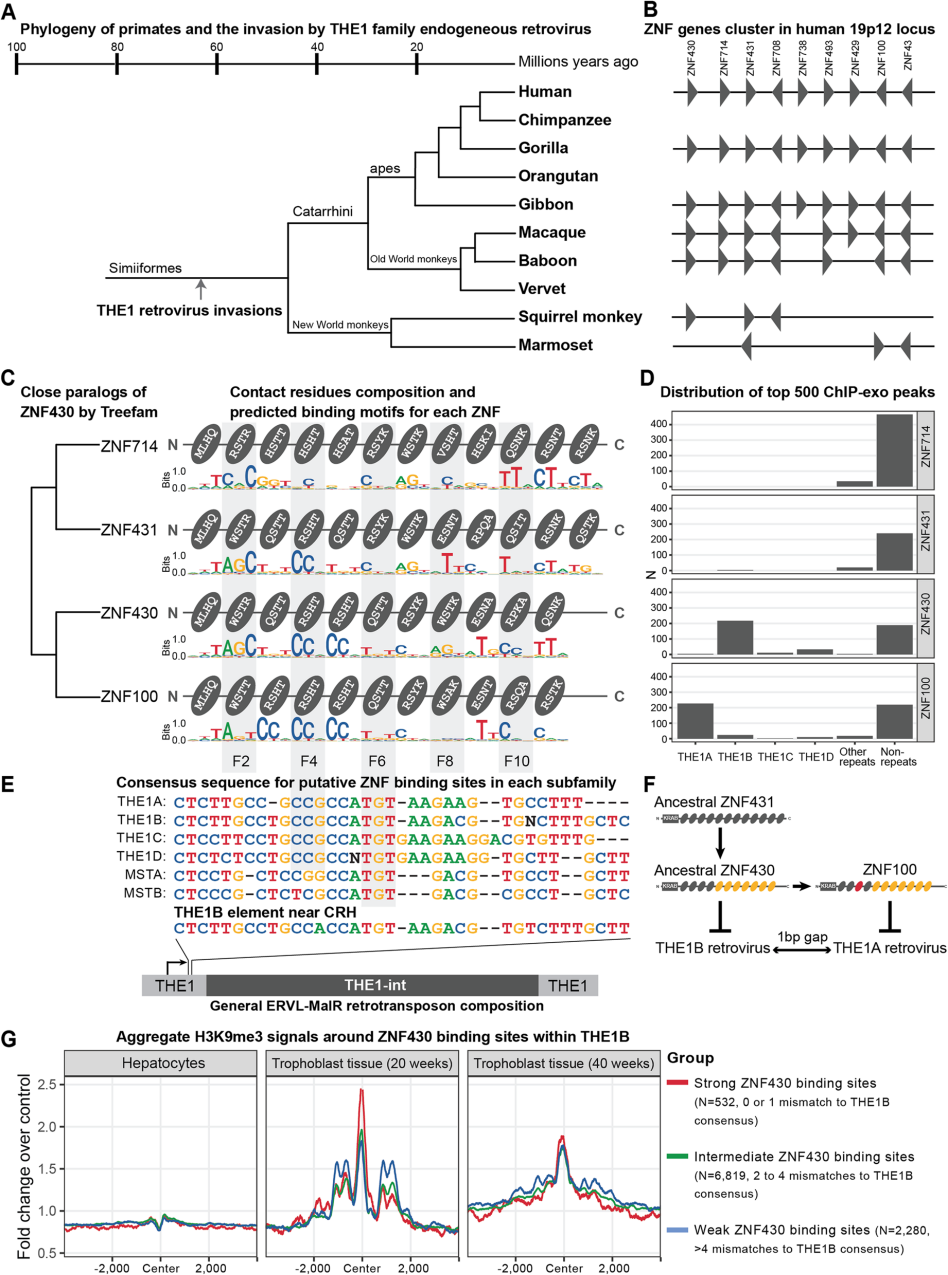
**A** Phylogeny of primates and the timing for the emergence of THE1 retrovirus; **B** ZNF genes cluster found in human 19p12 locus (only genes with human orthologs from ZNF430 to ZNF43 are shown); **C** Phylogeny of paralogs of ZNF430 according to Treefam (left panel); Contact residues by each finger and the predicted binding motifs by B1H methods, shown from N- to C- end (right panel); **D** Distribution of top 500 ChIP-exo peaks for each ZNF enriched in THE1 or other repeat elements (ZNF431 has fewer than 500 peaks in originally reported paper); **E** Consensus sequence for identified ZNF binding sites in each RVL-MalR retrotransposon; (Data source: Dfam); **F**  Proposed evolutionary history for ZNF430 and its paralogs. **G** Aggregate H3K9me3 signals around the putative ZNF430 sites within THE1B elements, sorted by mismatches to consensus, (Data source: ENCODE)

Large-scale analysis of human ZNFs ChIP-seq/exo data [4, 5] revealed that the peaks of ZNF430 and ZNF100 are strongly enriched within THE1B and THE1A retroelements respectively (Fig. 1D). Phylogenetic analysis of all human genes by Treefam [6] (Fig. 1C) show that, ZNF430, ZNF100, ZNF431, and ZNF714 are close paralogs located within the same 19p12 cluster (Fig. 1B), so most certainly they derive from one common ancestor through duplication and mutation processes. The co-existence of ZNF431/430/100 in New World Monkeys means these three genes emerged before the split of Catarrhines and New world monkeys. ZNF430 and ZNF100 are closer to each other than to ZNF431/714 (Fig. 1C, Fig. S2), so the two probably share the same ancestor. The identical contact residues in fingers 1–4 between ZNF431 and ZNF430 indicates both the ancestral ZNF431 and ancestral ZNF430 should have the same contact residues as current ZNF431/430 in fingers 1–4 regions. Also, ZNF431 is not expected to target any particular retrovirus, since its mouse ortholog was reported to be involved in the Hedgehog signaling [7].

MEME or RCADE analysis of ZNF430 and ZNF100 ChIP-exo data [5] (Fig. S4) pinpoint some 30nt long sequences as their specific binding sites within THE1B and THE1A respectively (Fig. 1E). In comparison, their closely related MSTA/B family retrovirus (Fig. S3) don’t contain the same sequences in corresponding loci and are thus not bound by ZNF430/100. Visual comparison between the B1H-predicted motifs of ZNF430/100 [8] and their consensus binding sites [9] reveals that, their fingers 4–6 are engaged in the recognition of CCGCCATGT sites (Fig. 1C, E). Moreover, the striking binding pattern difference between the two can be attributed to their contact residue changes and cognate binding sites difference, i.e., the finger three of ZNF100 uniquely recognizes the CCG site within THE1A elements. So taken together, THE1 family retroviruses invaded the Simiiformes more than 40 million years ago, and ZNF430 emerged to specifically silence the THE1B elements, then ZNF100 was evolved from the duplicated ZNF430 through limited codon changes (primarily F3 region) to repress the closely related THE1A elements. It is open question whether the 1 bp deletion within THE1A’s binding site contributed to its expansion (> 4,000 copies in human genome, Kimura Div. 8.4% [9]), because THE1A escaped silencing by ZNF430 and gained selective advantages over THE1B (~ 18k copies, Kimura Div. 10.2%) for certain periods of time before the emergence of ZNF100.

The identification for ZNF430/100 binding sites and recognition patterns helps us evaluate their contributions to THE1B repression in different tissues. According to Human Protein Atlas, ZNF430 is ubiquitously expressed in most types of human cells [10], including all three types of trophoblasts (Fig. S5). By extracting all putative ZNF430 binding sites from human THE1B elements, it is feasible to sort them into three classes based on the number of mismatches to the consensus sequence and plot the average H3K9me3 signals for each class. Significant H3K9me3 signals are observed around ZNF430 sites of THE1B in human trophoblast tissues at 20 and 40 weeks respectively, decreasing from strong to weak sites, whereas no peaks can be detected in hepatocytes at all (Fig. 1G), which is consistent with very low expression level of ZNF430 in hepatocytes (0.4nTPM). Some recently published data in human trophoblast stem cells [11] shows similar H3K9me3 enrichment around THE1B elements (Fig. S6). Overall, ZNF430 does contribute to the THE1B repression in human trophoblast tissue, particularly for those sites matching the consensus sequence well.

For the reported CRH-proximal THE1B element, it contains an intact ZNF430 binding site (Fig. 1E) and significant ChIP-seq/exo signals are observed around this site in multiple cell lines (Fig. S7), so under the constitutive ZNF430 repression in various tissues including placenta, no enhancer mark can be detected around this element. Neither THE1 nor ZNF430 exists in rodents, so mouse isn’t the ideal model organism to study the role of primate-specific retrovirus. Without ZNF430 ortholog in mice, the observation of upregulated CRH by THE1B in transgenic mice should not serve as evidence that this retrovirus is having a role in human pregnancy. Also it is notable that their predicted DLX3 binding sites (TAATGA, TGATAT) near THE1B don’t perfectly match the DLX3 motif (TAATTG) learned from in vitro experiment [1], thus further study of DLX3’s role in regulating CRH expression is desirable.

## Discussion

Without canonical enhancer mark, it is not impossible that THE1B functions as some non-canonical enhancer [12]. To prove or falsify this possibility, it is desirable to test whether the CRH expression level is altered upon the deletion of the reported THE1B element in relevant cells, like Frost et al. [11] and Yu et al. [13] reported for other retrovirus in human trophoblast stem cells. To test whether abnormal activation of THE1B or loss of function (LoF) of ZNF430 contributes to human pregnancy disorder, a phenotypic test on genome-edited non-human simian, such as macaque, is needed.

Besides ZNF430 and ZNF100, simian-specific ZNF766 was also implicated in the silencing of THE1 family retroviruses generally [4]. Recent large-scale GWAS studies [14] suggest that the LoF or mutations of ZNFs are associated with many human health conditions (Table S1), but it is unclear how many of them are results of abnormal activation of those repressed retrovirus. The species-specific nature of retroviruses and corresponding KZNF repressors in human genome requires us dissect their functions in suitable model system and interpret the results carefully, otherwise more time and resources would be wasted. As more high-quality data and better recognition models of ZNFs become available, we can decipher the human evolutionary genetics and their biomedical implications behind them.

## Supplementary Information

**Additional file 1.**

## Data Availability

All data used in this study are listed in Table S2 in Supplemental Information. The analysis workflow of ZNF430 ChIP-exo peaks distribution and H3K9me3 enrichment around THE1B are available in GitHub repository ZFPCookbook (subdirectory ZNF430 and ZNF100, DOI: 10.5281/zenodo.7711894). Briefly, it takes three steps to plot the aggregate H3K9me3 signals: (1) Extraction of all putative ZNF430 binding sites from THE1B elements annotated by RepeatMasker alignment file (hg38.fa.align, THE1B positions 197 to 222); (2) Sorting of all full-length ZNF430 binding sites based on their number of mismatches to consensus sequence into three classes (Figs. 1G, S6A); (3) Plotting aggregate H3K9me3 signals around each class of sites using the protocol provided by soGGi package [15] with distance Around parameter as 4000. The units of preprocessed signal tracks (FCC or CPM) are preserved.

## Abbreviations

ERV: Endogenous retrovirus
KZNF: KRAB-domain zinc finger genes
LoF: Loss of function
