# Supplementary material for: THE1B may have no role in human pregnancy due to ZNF430-mediated silencing
Source: Mob DNA. 2023 May 22;14:6. doi: 10.1186/s13100-023-00294-6 (PMC10204343; doi:10.1186/s13100-023-00294-6)
Supplement: Supplementary file 1 — Additional file 1. [file 13100_2023_294_MOESM1_ESM.pdf]

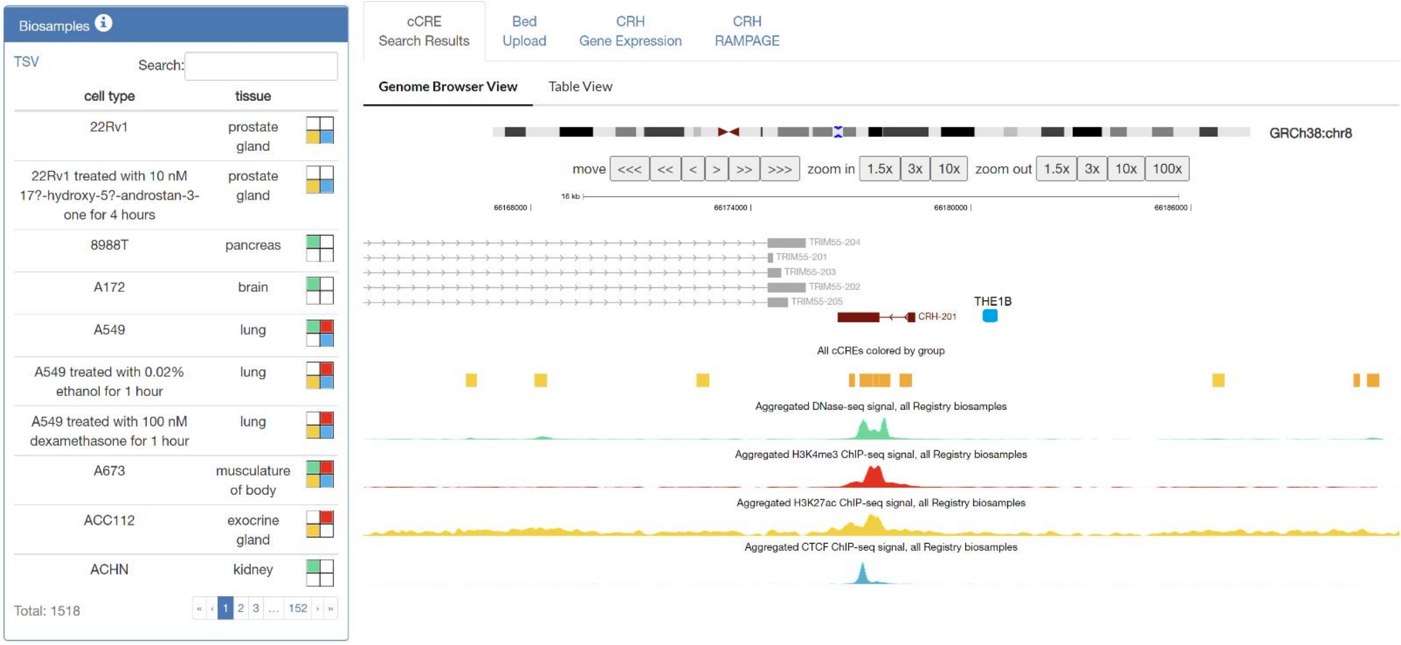


Figure S1 Absence of promoter or enhancer marks around THE1B element near CRH gene from ENCODE datasets.

(Source: https://screen.wenglab.org/)

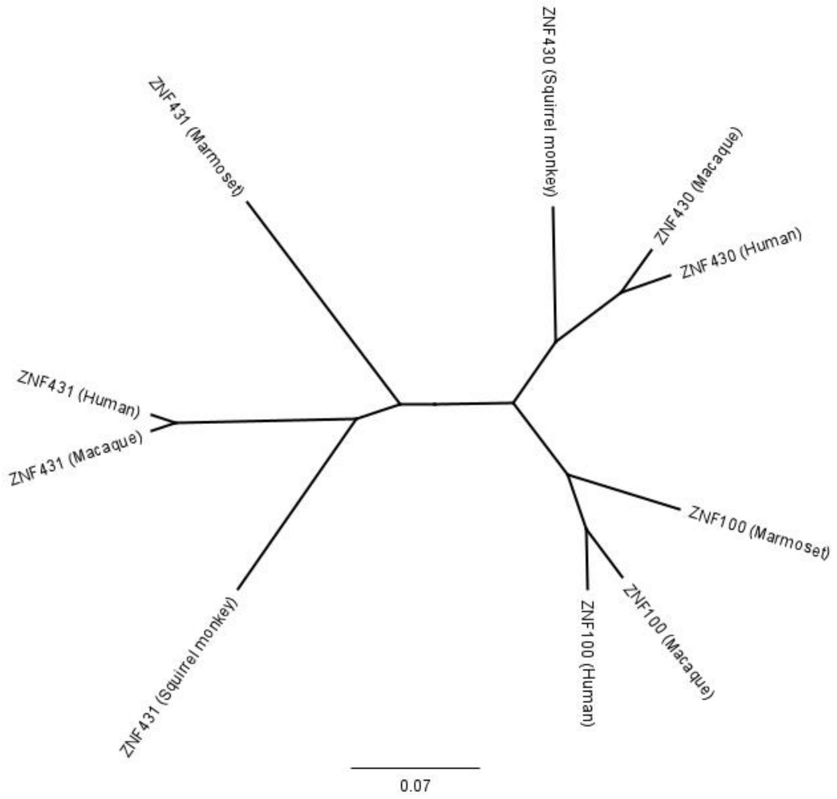


Figure S2 Phylogenetic tree of ZNF430 and its paralogs in some primates

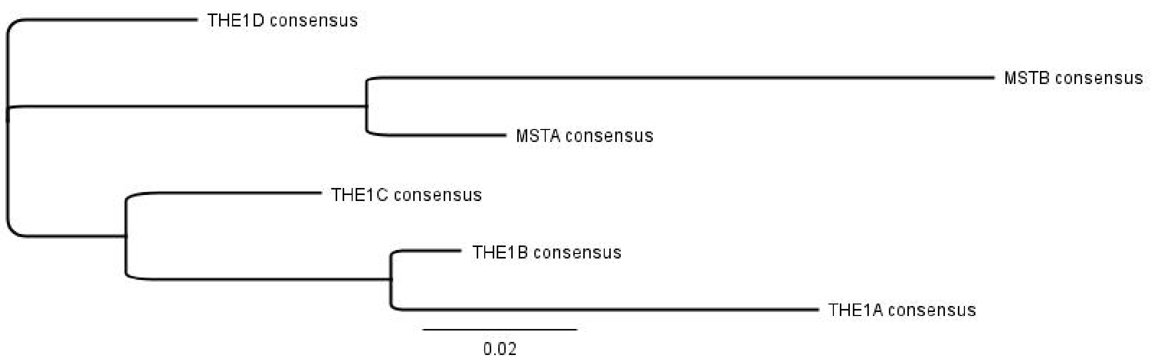


Figure S3 Phylogenetic tree of some ERVL-MalR family retrotransposons

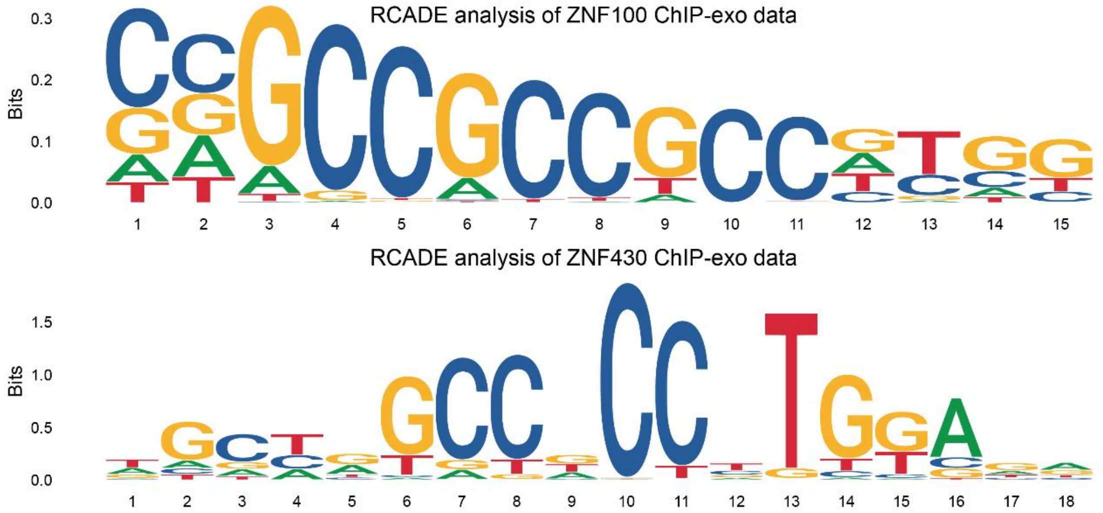


Figure S4 Motifs based on RCADE analysis of ChIP-exo data (source: CisBP)

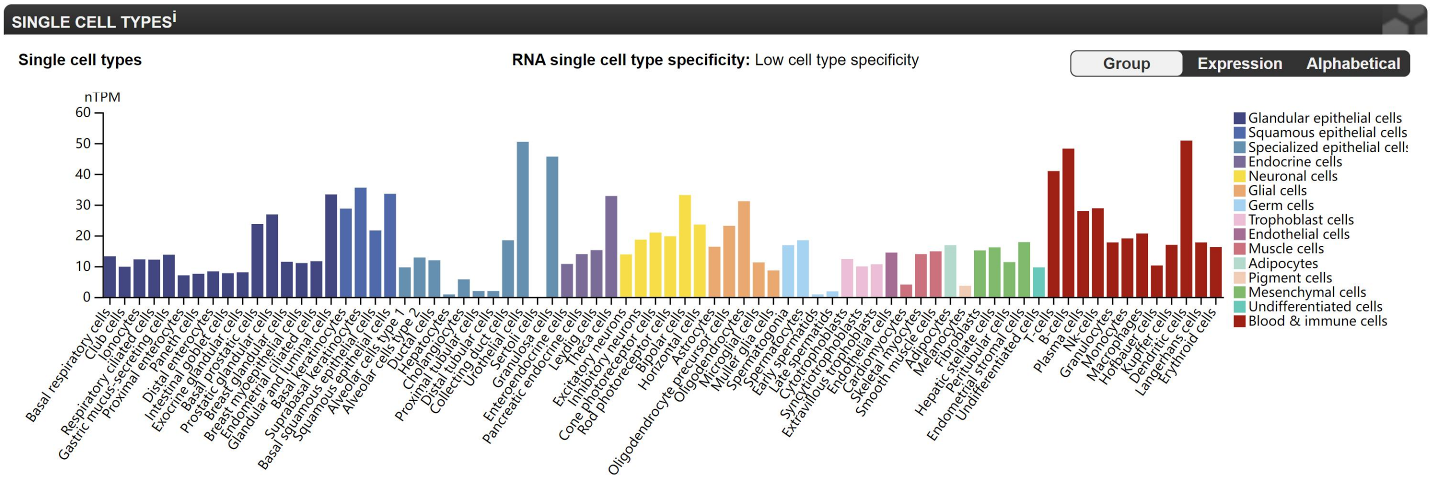


Figure S5 Expression levels of ZNF430 across different human cell types and tissues (Source: Human Protein Atlas)

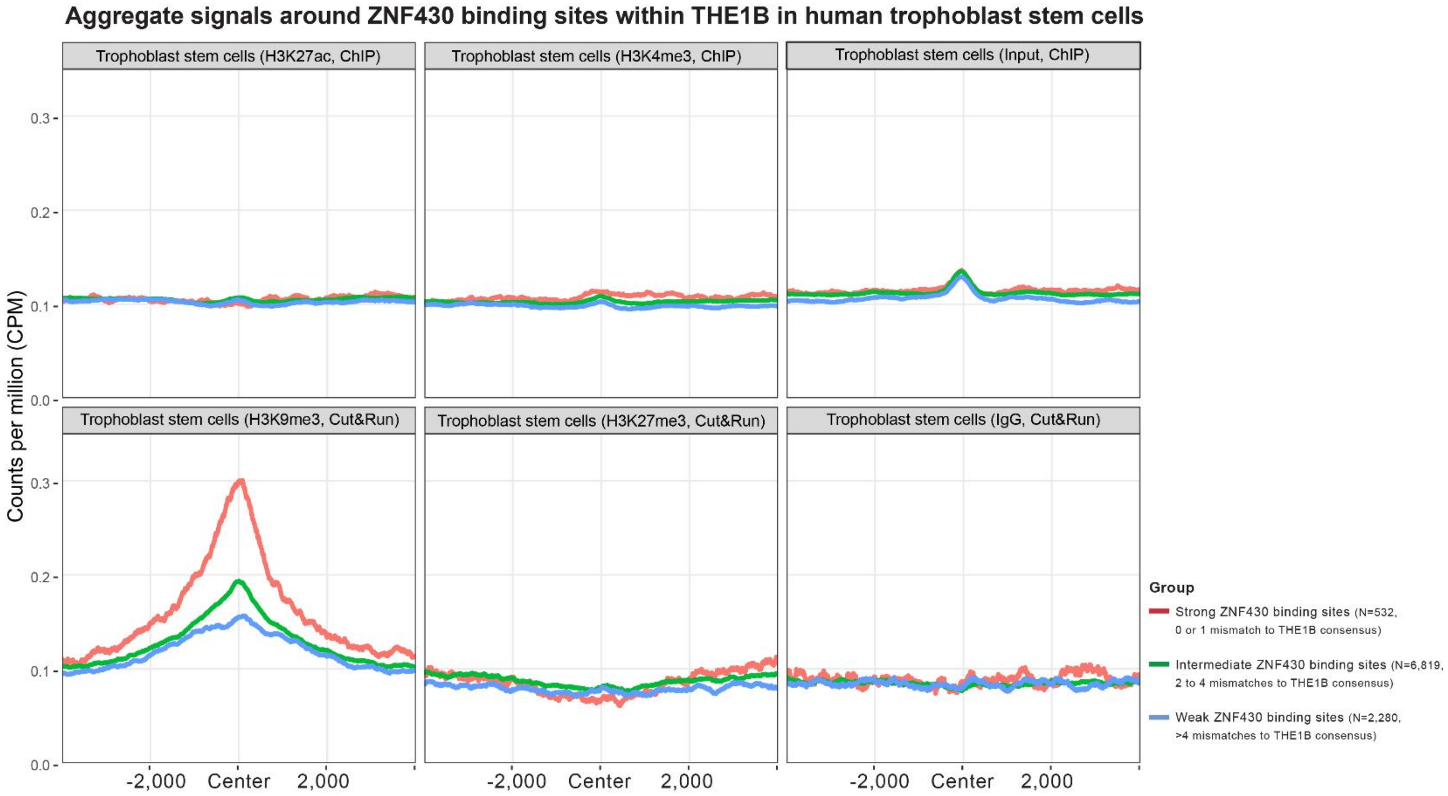


Figure S6 A) H3K9me3 signals are enriched around THE1B elements in human trophoblast stem cells.

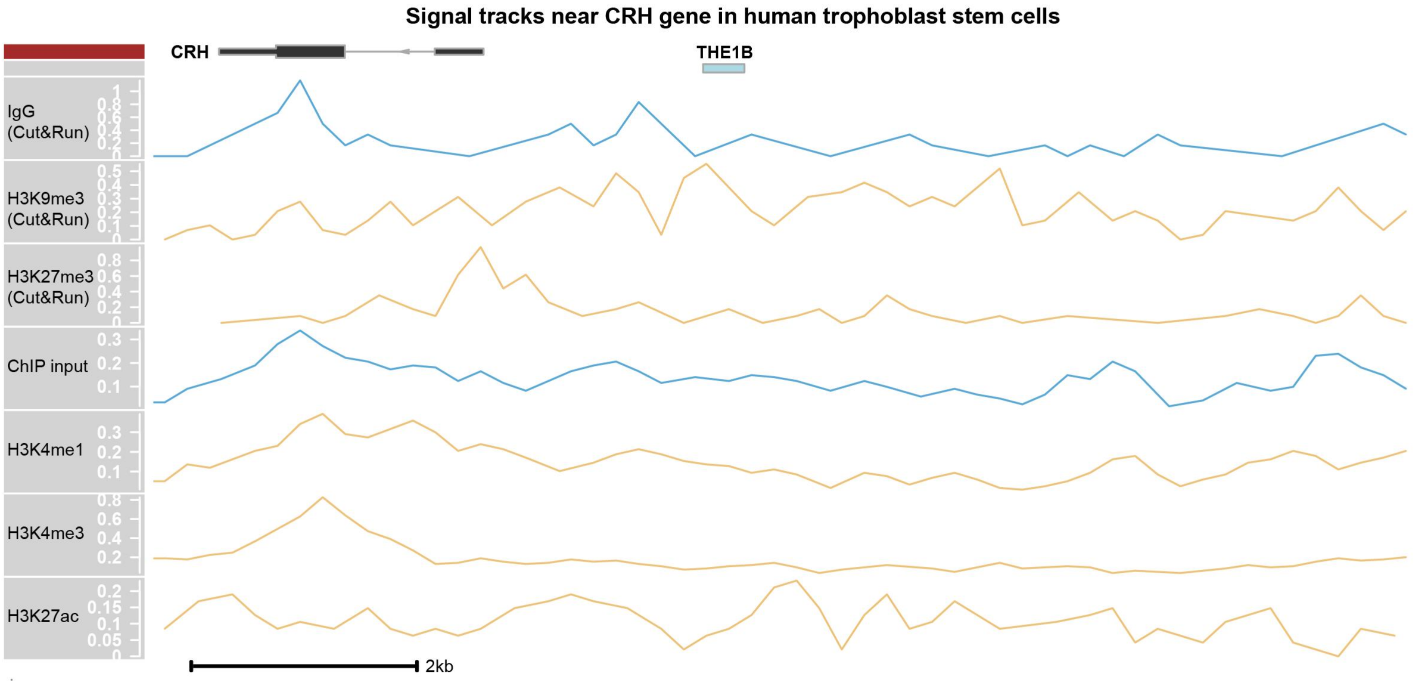


Figure S6 B) Various chromatin signal tracks near CRH gene in human trophoblast stem cells (Scale: Count per million (CPM); Data source: NCBI GEO GSE200763)

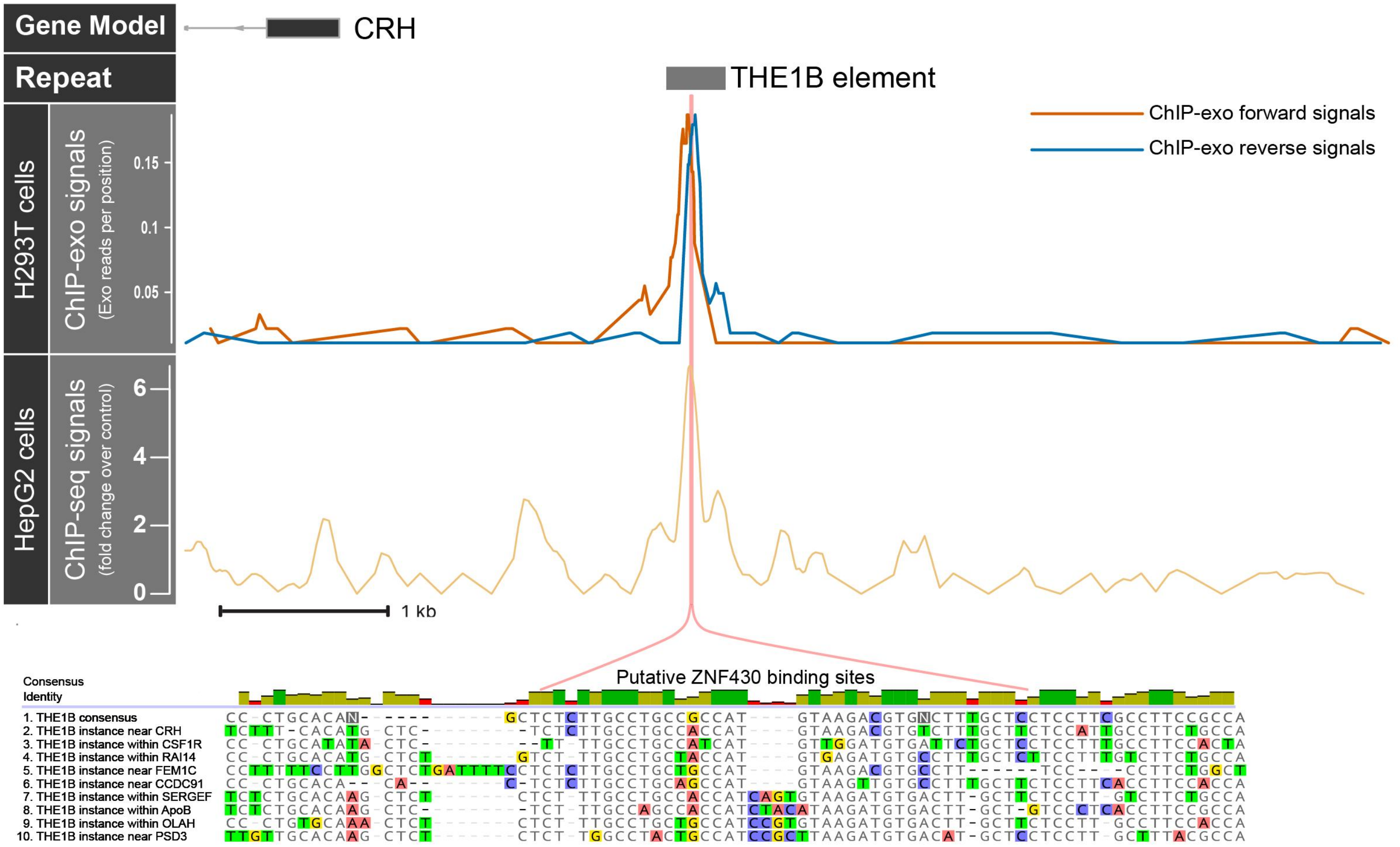


Figure S7 The ChIP-seq/exo signals tracks near human CRH promoter regions (hg38, chr8:66177800-66185000) and the putative ZNF430 binding sites within various THE1 elements.

| ZNF | Associated phenotype(s) | p-value (SKAT-O) | Disease allele frequency in population |
|---|---|---|---|
| ZSCAN31 | Hemoglobin concentration, Reticulocytes count | 1.44E-10 | 1.65E-01 |
| ZNF646 | Height | 1.17E-09 | 8.24E-03 |
| ZNF124 | Platelet count | 2.70E-08 | 6.88E-03 |
| ZNF467 | Platelet volume/Lymphocyte percentage | 1.48E-09 | 6.40E-03 |
| ZNF524 | Female BMI | 3.92E-08 | 6.25E-03 |
| ZNF417 | Mean platelet volume, platelet count | 2.15E-10 | 4.94E-03 |
| ZNF805 | Bone mineral density | 3.35E-07 | 3.66E-03 |
| ZNF222 | ApoB | 6.58E-11 | 3.64E-03 |
| ZNF518B | Urate | 4.40E-38 | 2.70E-03 |
| ZNF335 | High cholesterol | 5.70E-07 | 1.00E-03 |
| ZNF3 | Hemoglobin concentration/Red blood cells count | 1.28E-14 | 5.95E-04 |
| ZNF711 | Uterine polyps removed | 3.52E-08 | 1.00E-04 |
| ZNF316 | Mania/bipolar disorder/manic depression | 4.59E-06 | 1.00E-05 |
| ZFP14 | Depression | 2.20E-08 | 1.00E-05 |

Table S1 Association of human ZNF and various phenotypes (source: Genebass)

| REAGENT or RESOURCE | SOURCE | ACCESS IDENTIFIER |
|---|---|---|
| **Software, Algorithms, and Analysis** | | |
| MEME | MEME suite | http://meme-suite.org/ |
| Zinc finger motif prediction model | Singh Lab | zf.princeton.edu/ |
| ChIP-exo peaks distribution within Repeats and Aggregate H3K9me3 analysis | Zheng Zuo | https://github.com/zeropin/ZFPCookbook/tree/master/ZNF430%20and%20ZNF100 |
| RCADE analysis of ChIP-exo data | Hughes lab | http://kznfmotifs.ccbr.utoronto.ca/report.php?name=ZNF343 |
| **Zinc finger genes accession number** | | |
| ZNF431 (Squirrel monkey) | UCSC genome browser | JH378319.13 |
| ZNF430 (Squirrel monkey) | UCSC genome browser | JH378319.9 |
| ZNF100 (Marmoset) | UCSC genome browser | XP_035141469.1 |
| **Data source** | | |
| Zinc finger motif database | Hughes Lab | http://cisbp.ccbr.utoronto.ca |
| ChIP-exo data for ZNF430 and ZNF100 | Trono lab | NCBI GEO GSE78099 |
| ChIP-seq data for ZNF430 | ENCODE | ENCSR618END |
| Expression levels of ZNF430 | Human Protein Atlas | https://www.proteinatlas.org/ENSG00000118620-ZNF430/single+cell+type |
| ZNF genes association with phenotypes | GeneBass | Genebass.org |
| H3K9me3 ChIP-seq datasets | ENCODE | ENCFF073GYV, ENCFF040IBX, ENCFF509OEW |
| Enhancer screen from ENCODE database | ENCODE SCREEN | https://screen.wenglab.org |
| Chromatin signals in human trophoblast stem cells | Branco lab | NCBI GEO GSE200763 |

Table S2 Data sources and analysis workflow
